# FARS2 Mutations: More Than Two Phenotypes? A Case Report

**DOI:** 10.3389/fgene.2020.00787

**Published:** 2020-07-22

**Authors:** Mostafa Hotait, Wassim Nasreddine, Riyad El-Khoury, Maya Dirani, Omar Nawfal, Ahmad Beydoun

**Affiliations:** ^1^Department of Neurology, American University of Beirut Medical Center, Beirut, Lebanon; ^2^Department of Pathology and Laboratory Medicine, American University of Beirut Medical Center, Beirut, Lebanon

**Keywords:** FARS2, refractory status epilepticus, juvenile onset epilepsy, mitochondrial tRNA synthetase, mitochondrial epilepsy

## Abstract

FARS2, a nuclear gene, encodes the mitochondrial phenylalanyl-tRNA synthetase (mtPheRS). Previous reports have described two distinct phenotypes linked to FARS2 gene mutation: an early onset epileptic encephalopathy and spastic paraplegia. This report describes a distinctive phenotype of FARS2-linked, juvenile onset refractory epilepsy, caused by a hemizygous mutation in a compound heterozygous state (p.V197M and exon 2 microdeletion). A 17-year- old woman with normal development presented with a super refractory focal motor status epilepticus. Only an emergency life-saving surgery aborted her status after all therapeutic interventions, including anesthesia, failed to control her seizures. Pathological and biochemical activities on muscle biopsy showed mitochondrial proliferation with enhanced isolated activities of complexes II and IV, suggestive of a compensatory mechanism for the bioenergetic deficiency. Postoperatively, the patient started experiencing focal aware motor seizures originating from the contralateral hemisphere after being seizure free for a few months. This report suggests a third phenotypic manifestation of FARS2 gene mutation.

## Introduction

Mitochondria are extremely important organelles in cellular energy generation through the oxidative phosphorylation system (OXPHOS) exploiting the electron transport chain (ETC) complexes (Vantroys et al., 2017). Mitochondrial diseases refer to any defect in those complexes or their function. Seizures have been reported in up to 60% in patients with mitochondrial diseases and mitochondrial epilepsy is commonly characterized by the occurrence of epilepsia partialis continua soon after the onset of focal epilepsy (Rahman, 2012). Although mitochondria contain their own DNA (mtDNA), 99% of the mitochondrial proteins are encoded by nuclear genes, including the majority of ETC complexes subunits, and proteins implicated in transcription and translation among others (El-Hattab et al., 2017). The mitochondrial amino-acyl tRNA synthetases (mt-aaRSs) are a well-described group of enzymes that play a key role in mitochondrial protein biosynthesis and responsible for catalyzing the attachment of each of 20 amino acids to their cognate mitochondrially encoded tRNAs (Konovalova and Tyynismaa, 2013). Most of the mitochondrial and cytoplasmic aaRSs are encoded by nuclear genes (Konovalova and Tyynismaa, 2013). Defects in any of the mt-aaRSs can result in a defective intramitochondrial translation, affecting the OXPHOS complexes containing mitochondrially encoded subunits (complexes I, III, IV, and V). Recently, various mitochondrial diseases have been linked to pathogenic variants in nuclear genes encoding mitochondrial aminoacyl-tRNA synthetases. FARS2, a nuclear gene located on chromosome 6 (6p25.1), encodes the mitochondrial phenylalanyl-tRNA synthetase (mtPheRS), which transfers phenylalanine (Phe) to its cognate tRNA in mitochondria. The first pathogenic variant of FARS2 gene was reported in 2012 in a 2-year-old girl with developmental delay, seizures, myoclonus, and lactic acidosis (Shamseldin et al., 2012). Since that time, two distinct phenotypes, consisting of early onset epileptic encephalopathy and spastic paraplegia have been linked to FARS2 gene mutations. We report a patient with juvenile onset refractory focal onset epilepsy and the presence of a third distinctive phenotypic manifestation of FARS2 gene mutation.

## Case Report

**FIGURE 1:**
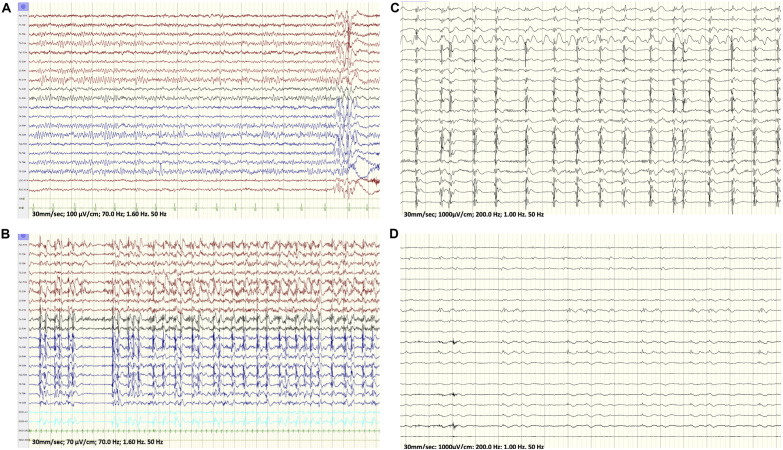
**(A)** Baseline EEG at onset of her seizures showing a generalized atypical spike and wave discharges with a right sided predominance. **(B)** EEG: While maintained on propofol, seizures originated from the right hemisphere out of a burst-suppression pattern. **(C,D)** Intraoperative electrocorticograph. **(C)** Prior to resection. **(D)** Post right insula and right prefrontal resection and right face motor subpial transaction.

**FIGURE 2:**
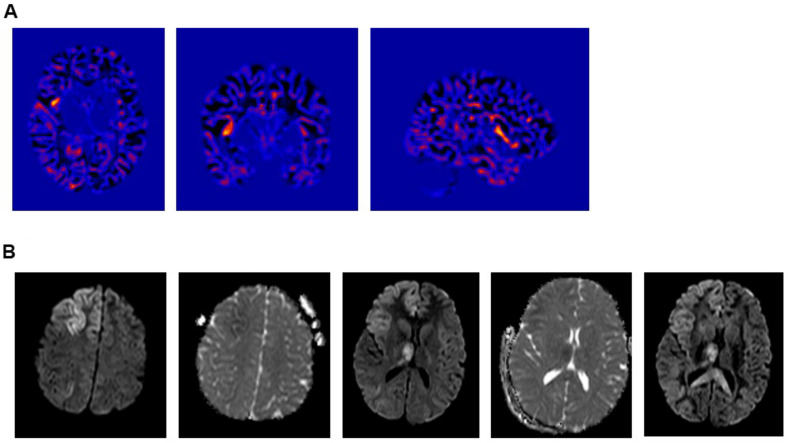
**(A)** Voxel Based Morphometric (VBM) Analysis – Junction Map. (i) Axial; (ii) Coronal, and (iii) Right Sagittal cuts. Showing a suspicious lesion involving the right anterior insula with a high Z-core. **(B)** Intra-ictal brain MRI diffusion weighted images (DWI) showing a restricted diffusion in the cortical-subcortical area of the right frontal lobe, right insula and right thalamus.

The patient is a 17-year-old right-handed girl, born to unrelated parents with a normal neurodevelopment and no risk factors for epilepsy who presented with focal motor aware status epilepticus. Her history goes back to 1 year prior to presentation when she started to experience rare brief focal aware clonic seizures semiologically characterized by twitching of the left side of the face. On a combination of lamotrigine and levetiracetam, her opercular seizures were relatively well controlled with a breakthrough seizure every 1 to 2 months. Her workup included an epilepsy protocol MRI that was normal and a 3 h video/EEG recording, which revealed a normal posterior background and fragments of generalized spike-wave discharges (GSWDs) with a right sided amplitude predominance, thought to represent an inherited EEG trait (Figure 1A). A post-acquisition morphometric analysis of the brain MRI revealed a suspicious lesion in the right insula based on a high z score on the junction map (Figure 2A). Moreover, a volumetric brain analysis revealed no volume difference between the right and left gray matter structures and did not show any global atrophy or asymmetry between the two hemispheres.

On the day of her admission, the patient presented to the emergency department (ED) in focal aware motor status epilepticus of 2 days duration with seizures characterized by left gaze deviation associated with left facial twitches and left upper extremity clonic jerking. Her vital signs were stable and her physical and neurological examinations were otherwise unrevealing. The patient was monitored with continuous video/EEG that revealed near continuous seizures originating from the right fronto-central area. Over the subsequent 24 h, the seizures persisted despite treatment with levetiracetam, lamotrigine, valproate, and lacosamide.

Cerebrospinal fluid (CSF) revealed no white cell counts, normal levels of protein and glucose, negative culture and negative meningitis panel. Complete blood count (CBC), lactic acid and hepatic enzymes were normal and blood for whole exome sequencing (WES) was drawn. Pulse treatment with methylprednisolone administered for a possible autoimmune encephalitis failed to abort her seizures. At that time, the patient became progressively obtunded, continued to experience intermittent subtle left facial twitches and developed paresis of her left upper extremity. Her EEG continued to show very frequent seizures that were wider in distribution and involved the left hemisphere. A repeat brain MRI showed restricted diffusion in the cortical-subcortical areas of the right frontal lobe, right insula, right thalamus and to lesser extent in the right temporal, both parietal lobes and left frontal lobe (Figure 2B). Because of the persistence of the status epilepticus and the EEG findings, in conjunction with the change in the patient’s mental status, the patient was intubated and treatment with anesthetic was initiated. The seizures failed to respond to high dose midazolam infusion and the patient developed hemodynamic compromise on propofol with no control of the electrographic seizures that persisted despite reaching a burst-suppression on the EEG (Figure 1B).

Considering the clinical deterioration of the patient and the refractory status epilepticus, it was decided to operate on the patient focusing on the right opercular area and the suspicious area in the right insula identified on brain morphometric analysis. Intra-operatively, the brain was edematous with right frontal lobe hyperemia and evidence of increased blood flow. Intraoperative electrocorticography revealed nearly continuous discharges from the right centrofrontal head region (Figure 1C) that eventually nearly disappeared following a right insular and prefrontal resection along with subpial transections over the right face motor area (Figure 1D). Histologic analysis of the resected prefrontal cortex revealed heterotopic neurons in addition to hypoxic/ischemic changes.

Postoperatively, patient experienced a transient left central facial palsy and left distal upper extremity weakness. Her EEG at that time revealed rare focal epileptiform discharges originating from the right centroparietal head region in addition to fragments of GSWDs. She remained seizure free for 3 months post-operatively then started to experience focal aware clonic seizures from the contralateral hemisphere, semiologically characterized by right gaze deviation and right hemiclonic seizures.

Whole exome sequencing revealed a novel hemizygous mutation in FARS2 gene in a compound heterozygous state, with a deletion of exon 2 in one of the parent copies (confirmed by qPCR) and a Val197Met mutation in exon 2 of the second copy, confirmed by Sanger sequencing (Supplementary Figure 1). The length of the detected deletion comprised 612-bp, starting at 5368803 and ending 5369415, which corresponded to a deletion of the entire exon 2 of the FARS2 gene. According to the ACMG guidelines, the microdeletion copy number variation was classified as a likely pathogenic mutation (Class 2) and the substitution mutation was classified as a variant of uncertain significance (Class 3). WES and Sanger sequencing were performed and validated by the Centogene lab.

**FIGURE 3:**
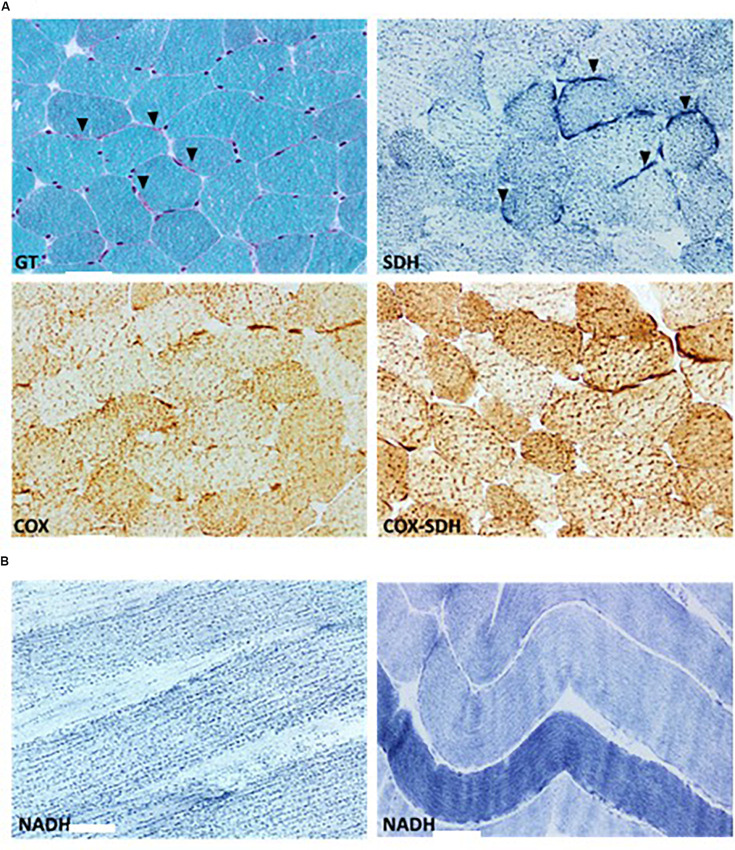
Histological assessment of the patients’ muscle biopsy. **(A)** Modified Gomori’s Trichrome (GT) stain outline general features characterized by subsarcolemmal mitochondrial proliferation (arrowhead) and multifocal clusters of disintegrating myofibers. Succinate dehydrogenase (SDH) and cytochrome c oxidase (COX) histoenzymatic stains illustrate normal SDH and COX activities and highlight the reactive subsarcolemmal mitochondrial proliferation (arrowhead). COX negative fibers are absent as indicated by lie COX-SDH overlay stain. **(B)** NADH histoenzymatic reaction demonstrate lie diffuse loss of mitochondrial network and marked mitochondrial aggregation (left) compared to an age matched control (right). Upper and lower panels represent 400-fold magnification of sections. Scale bars: 50 μm.

A muscle biopsy was performed to evaluate the impact of this pathogenic mutation on mitochondrial pathology and respiratory chain function. Pathological studies of the skeletal muscle showed mild variation in myofibers size, minimal fibrosis and multifocal clusters of disintegrating myofibers. Intramyocellular lipid globules were excessively increased both in size and density. Histochemical assessment of oxidative enzymes, including NADH-tetrazolium reductase, succinate dehydrogenase and cytochrome c oxidase (COX), showed normal activities (Figure 3A). ATP synthase showed a normal expression on immunohistochemical stain. Reactive subsarcolemmal mitochondrial proliferation was frequently observed (Figure 3A). Mitochondrial network fragmentation with excessive mitochondrial aggregation was diffuse (Figure 3B). No ragged red fibers (RRFs) and no COX negative fibers were noted.

Quantitative evaluation of mitochondrial respiratory chain complexes activities was assessed spectrophotometrically on a homogenate prepared from a frozen fragment of the patient’s muscle biopsy. Isolated activities of complexes I (NADH dehydrogenase), III (cytochrome *bc*_1_), and V (ATP synthase) were within their normal ranges, while isolated activities of complexes II and IV were increased (Table 1). Combined complexes II+III activity was also above its normal limits. Mitochondrial mass was preserved as indicated by the normal activity of the reference enzyme citrate synthase. Normalized activities, expressed in terms of the citrate synthase level for each individual complex, were within their normal reference ranges for all complexes. Cytosolic lactate dehydrogenase activity was normal.

**TABLE 1:** Biochemical analysis of mitochondrial respiratory chain complexes activities.

	Activities (nmol.min^–1^.mg^–1^)	Ref. range (nmol.min^–1^.mg^–1^)

	Patient	
**CI**	20.91	[10.42–47.30]
**CII**	54.36*	[18.73–47.70]
**CIII**	121.97	[81.28–210.86]
**CIV**	264.79*	[82.01–237.59]
**CV**	105.28	[62.21–130.72]
**CII+III**	45.79*	[16.21–33.24]
**CS**	226.23	[110.86–288.65]
**LDH**	5448	[2084–7317]

Spectrophotometry-based assays were used in order to analyze isolated enzymatic activities of NADH dehydrogenase (CI), succinate dehydrogenase (CII), coenzyme Q cytochrome c-oxidoreductase (CIII), cytochrome c oxidase (CIV) and ATP synthase (CV) as well as combined CII+III activities. Citrate synthase (CS) activity was used as a control for mitochondrial mass, while lactate dehydrogenase (LDH) was used as a cytosolic reference. Normalized activities, expressed in terms of the citrate synthase level for each individual complex were within normal ranges in both cases. Values represent enzymatic activities and are expressed in nanomoles of substrate transformed per minute per milligrams of proteins (nmol.min^–1^.mg^–1^). *indicates activities that do not fall within our normal reference values.

The patient is now 14 months since her operation with no evidence of psychomotor regression. She is experiencing a cluster of 2–3 focal aware clonic seizures every 6 weeks, originating from the left frontocentral area while maintained on a combination of levetiracetam, lacosamide, phenobarbital and clonazepam.

## Discussion

In this report, we present a novel hemizygous mutation in a compound heterozygous state with a deletion of exon 2 in one of the parent copies and a Val197Met mutation in exon 2 of the second copy, which was previously reported only once as part of a compound mutation (Chen and Zhang, 2019). This missense mutation has been analyzed as disease causing using Polyphen-2 with a reported prevalence of 3/119,738 (Chen and Zhang, 2019).

Since FARS2 gene mutation was first reported in 2012 (Shamseldin et al., 2012), a total of 37 additional cases were reported. Two distinct clinical phenotypes consisting of an early onset epileptic encephalopathy and a late onset spastic paraplegia were recognized with the vast majority of FARS2 mutations (35/37) fitting one of those two phenotypes (Table 2; Almannai et al., 2019). The early onset epileptic encephalopathy is characterized by seizures with an onset in the first 6 months of life that rapidly become intractable with frequent episodes of status epilepticus (Vernon et al., 2015; Raviglione et al., 2016; Cho et al., 2017; Almannai et al., 2018). In addition, the affected children have profound developmental delay, lactic acidosis, evidence of liver dysfunction and typically die before the age of 2 years (Table 2; Almannai et al., 2019). Most of the early-onset epileptic encephalopathy are associated with the pathogenic variant (c.431A > G: p.Y144C) affecting the catalytic domain and were predominantly reported in patients from Arab descent (Almannai et al., 2018). The late onset phenotype mainly manifests with a spastic paraplegia with an onset within the first 5 years of life. Although 30% of children with the late phenotype experience seizures, those are typically mild and self-limited (Table 2; Almannai et al., 2019).

**TABLE 2:** Clinical, biochemical, and radiological features of FARS2 related disorders.

	Early onset phenotype	Late onset phenotype	Juvenile onset phenotype
Number of subjects	26	10	3
Consanguinity	54%	50%	0
Age at presentation	Birth-6 months	1 months-5 years	8–17 years
Outcome (Alive)	35%	100%	33%
**Neurological manifestation**			
Developmental delay	24/24 (100%)	6/10 (60%)	1/3 (motor and speech)
Truncal hypotonia	16/19	2/9	0/3
Peripheral spasticity	11/19	10/10	0/3
Seizures	24/25	3/10	3/3
**Neuroimaging**			
Brain atrophy	15/19	2/10	2/3
**Metabolic disease**			
Hepatic disease	14/19	0/10	0/3
Failure to thrive	9/17	0/8	0/3
**ETC complexes**			
Low complex I-IV activity	4/7	1/2	0/2
Normal activity	2/7	0/2	1/2*

*Enzyme activities in our patient were neither normal nor low but rather increased, more specifically Complexes II and IV.

Our patient represents a distinctive phenotype of FARS2 gene mutation with a seizure onset at the age of 16 years, in an otherwise developmentally normal adolescent. She started by experiencing focal aware clonic seizures, with the symptomatogenic zone initially localized to the right opercular area. Her seizures remained relatively well controlled for a period of 1 year before developing super-refractory status epilepticus. There are only two recent reports describing a FARS2- linked, juvenile onset epilepsy (Walker et al., 2016; Chen and Zhang, 2019). The first case is of a girl with motor and speech delays noted during the first few years of life who developed her first generalized tonic-clonic seizure at the age of 8 years (Walker et al., 2016). Her seizures remained poorly controlled until the age of 13 years when she developed refractory focal motor status epilepticus for which she underwent a corpus callosotomy. Her symptoms then progressed to a global severe cognitive deterioration associated with near constant multifocal myoclonus that persisted until her death at the age of 15 years (Walker et al., 2016). WES performed on this patient identified compound heterozygous mutations in FARS2: c.253C > G (p.P85A, exon 2) and c.403C > G (p.H135D, exon2) (Walker et al., 2016). The second case is that of a developmentally normal young man with a history of generalized tonic-clonic seizures with an onset at 12 years of age that remained well controlled until the age of 17 years when he presented with focal to bilateral tonic-clonic seizures that progressed to generalized convulsive status epilepticus eventually controlled with anesthetic agents (Chen and Zhang, 2019). His seizures subsequently remained poorly controlled and the patient died at the age of 20 years from pulmonary failure following a respiratory infection (Chen and Zhang, 2019). DNA sequencing revealed two novel compound heterozygous mutations in FARS2 gene, c.589G > A (p.V197M) and c.1205T > C (p.F402S) (Chen and Zhang, 2019).

Broad histochemical studies of our patient’s muscle biopsy showed organizational and functional mitochondrial impairment characterized by diffuse mitochondrial network disruption and excessive aggregation of mitochondria, in addition to subsarcolemmal proliferation of mitochondria. Those pathological findings most likely denote a bioenergetic deficiency triggering a reactive proliferation of mitochondria as an early defense mechanism in order to compensate for the ongoing structural disintegration as indicated by our histological studies. This compensatory mechanism of mitochondrial proliferation was also reported in two other patients with FARS2 mutation (Almannai et al., 2019). The observed enhanced biochemical activities of complexes II, IV, and II+III further highlights the compensatory mechanism to dysfunctional mitochondria. Previously, ETC complexes activity was reported to be normal except for some subjects whose biochemical studies showed evidence of a slight reduction in the activity of complexes I – IV (Walker et al., 2016; Almannai et al., 2019). Excessive intramyocellular lipid deposits, further denotes the mitochondrial impairment, triggering a disruption of mitochondrial fatty acids beta-oxidation.

Our patient underwent an emergency epilepsy surgery as a life-saving operation because of the progressive hemodynamic deterioration and worsening of the electrographic seizures despite anesthetic treatment. In a previous case, a corpus callosotomy was attempted to treat the refractory epilepsia partialis continua but without success (Walker et al., 2016). In our case, the prefronto-insular resection guided by intraoperative electrocorticography resulted in seizure freedom for a period of 3 months. The focal aware clonic seizures then only recurred from the contralateral hemisphere but are so far relatively well controlled and since her surgery, 14 months ago, there is no clinical evidence of cognitive deterioration.

Our case in conjunction with the two previous reports strongly suggests the presence of a third phenotype associated with FARS2 mutations consisting of focal aware motor seizures sometimes progressing to bilateral tonic-clonic seizures appearing during late childhood or adolescence (Table 2). Those seizures are initially relatively well controlled on AED treatment for a few years before evolving to a super refractory status epilepticus. Subsequent to the episode of status, there appear to be a downhill course with refractory seizures and death a few years later from recurrent infections (Walker et al., 2016; Chen and Zhang, 2019). This clinical presentation is reminiscent of that of Alpers-Huttenlocher disease (AHD), which has a bimodal age at seizure onset, the most common being between the ages of 2–4 years and the second during adolescence and early adulthood (Lim and Thomas, 2020). The most common presentation of POLG related disorders consist of focal motor seizures with frequent progression to epilepsia partialis continua. Other seizure types include focal seizures of occipital origin as well as myoclonic seizures (Lim and Thomas, 2020). A characteristic EEG pattern known as rhythmic high-amplitude delta with superimposed (poly) spikes is suggestive of this diagnosis (van Westrhenen et al., 2018). In that regard, it is interesting to note that a few of the reported FARS2 mutations fulfilled the criteria of Alpers-Huttenlocher disease (AHD) including the neuropathological findings (Elo et al., 2012; Konovalova and Tyynismaa, 2013). Our findings in conjunction with the other two cases expand the phenotypic expression of FARS2 mutations and indicate that in patients presenting in late childhood or early adolescence with focal seizures progressing to medical refractoriness, FARS2 gene mutation should be considered in the differential diagnosis.

## Data Availability Statement

The raw data supporting the conclusions of this article will be made available by the authors, without undue reservation.

## Ethics Statement

Ethical review and approval was not required for the study on human participants in accordance with the local legislation and institutional requirements. The patients/participants provided their written informed consent to participate in this study. Written informed consent was obtained from the patient for the publication of any potentially identifiable images or data included in this article.

## Author Contributions

MH and WN were responsible for the data collection and interpretation, literature review, and wrote the initial draft of the manuscript. RE-K did all the histopathology and biochemical analysis of the muscle biopsy. MD and ON followed the patient clinically and participated in writing the initial draft. AB edited and wrote the final version of the manuscript. All authors contributed to the article and approved the submitted version.

## Conflict of Interest

The authors declare that the research was conducted in the absence of any commercial or financial relationships that could be construed as a potential conflict of interest.
