# Supplementary figures and images for: FARS2 Mutations: More Than Two Phenotypes? A Case Report
Source: Front Genet. 2020 Jul 22;11:787. doi: 10.3389/fgene.2020.00787 (PMC7387725; doi:10.3389/fgene.2020.00787)

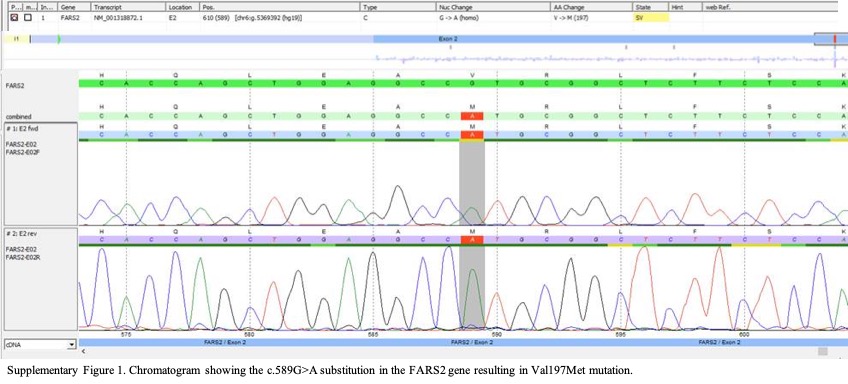

Supplement: Supplementary file 1 [file Image_1.JPEG]
